# Application of low-concentration contrast agents and low-tube-voltage computed tomography to chest enhancement examinations: A multicenter prospective study

**DOI:** 10.1177/0036850419892193

**Published:** 2019-12-02

**Authors:** Donghua Meng, Xiaonan Cui, Changsen Bai, Zhongwen Yu, Lei Xin, Yufei Fu, Sicong Wang, Yu Du, Zhipeng Gao, Zhaoxiang Ye

**Affiliations:** 1 Department of Radiology, Tianjin Medical University Cancer Institute and Hospital, National Clinical Research Center for Cancer, Key Laboratory of Cancer Prevention and Therapy, Tianjin’s Clinical Research Center for Cancer, Tianjin, China; 2 Department of Laboratory, Tianjin Medical University Cancer Institute and Hospital, National Clinical Research Center for Cancer, Key Laboratory of Cancer Prevention and Therapy, Tianjin’s Clinical Research Center for Cancer, Tianjin, China; 3 Department of Radiology, China Resources Wuhan Iron and Steel General Hospital, Wuhan, China; 4 Department of Radiology, Shanxi Cancer Hospital, Taiyuan, China; 5 Department of Radiology, Edong Medical Group Central Hospital, Huangshi, China; 6 GE Healthcare Life Science, Beijing, China; 7 Department of Radiology, The Fourth Hospital of Hebei Medical University, Shijiazhuang, China

**Keywords:** Multicenter, contrast agent, tube voltage, computed tomography, chest

## Abstract

To evaluate the influence of low-concentration contrast agents and low-tube-voltage computed tomography on chest enhancement examinations, we conducted a multicenter prospective study. A total of 216 inpatients enrolled from 12 different hospitals were randomly divided into four groups: A: voltage, 120 kVp; iohexol, 350 mgI/mL; B: voltage, 100 kVp, iohexol, 350 mgI/mL; C: voltage, 120 kVp, iodixanol, 270 mgI/mL; and D: voltage, 100 kVp, iodixanol, 270 mgI/mL. Subjective image quality was assessed by two radiologists and compared by weighted kappa test. The objective image scores, scanning radiation doses, and pathological coincidence rates were analyzed. There were no significant differences in gender, age, height, weight, and body mass index between the four groups (p > 0.05). The consistency of the radiologists’ ratings were good, with kappa value ranging from 0.736 (95% confidence interval: 0.54–0.933) to 0.809 (95% confidence interval: 0.65–0.968), and there was no difference in subjective image score between the four groups. The computed tomography value of group D had no difference with group A. The volume computed tomography dose index, dose length product, and effective dose of group D (6.93 ± 3.03, 241.55 ± 104.75, and 3.38 ± 1.47, respectively) were all significantly lower than those of group A (10.30 ± 4.37, 359.70 ± 152.65, and 5.04 ± 2.14, respectively). There was no significant difference in the imaging diagnosis accuracy rate between the four groups (p > 0.05). The results indicated that low-concentration contrast agents (270 mgI/mL) and low-tube-voltage (100 kVp) computed tomography can not only decrease radiation dose but also guarantee the image quality and meet the needs of imaging diagnosis in chest enhancement examinations, which make it possible for its generalization and application.

## Introduction

Multidetector row computed tomography (CT) imaging is widely used as the valuable imaging method for diagnosing lung diseases.^1,2^ It can make a clear judgment on the size, shape, density, and location of the disease and has become an indispensable imaging mode in a wide range of clinical applications.^3,4^ However, in some cases, routine CT examinations do not meet diagnostic needs.^5–7^ For example,^8^ as early lesions of lung adenocarcinoma, preinvasive and minimally invasive adenocarcinoma lesions are less invasive to local structures, and many common signs of lung adenocarcinoma are rarely seen in these lesions (burrs, lobes, empty bubbles, and pleural depression). The fact that the CT values between ground glass opacity and surrounding normal lung tissue are not very high may complicate the assessment of some common signs. Preoperative diagnosis based on traditional morphological findings is very difficult. At this time, it is necessary to use enhanced CT to analyze the lesion to obtain an accurate preoperative diagnosis.

Enhanced CT examination through the body injection of contrast agents can clearly show the blood supply of space-occupying lesions, as well as a good and malignant judgment of its pathological properties, providing an important basis for the determination of the next treatment.^9–11^ However, as the number of enhanced CT scans in the lungs increases, the radiation dose problem has become a focus of attention.^12^ How to reduce the scanning dose is a clinical hot spot, and it is difficult without affecting the quality of CT images. It is also the goal of various instrument manufacturers and users.^13,14^ In the past 10 years, more people have begun to focus on optimizing the radiation dose and safety of contrast agents of chest-enhanced CT scans, aiming to achieve CT images that meet clinical diagnostic needs with minimal radiation doses and safer contrast agents.^15,16^ For reducing radiation exposure, technicians usually reduce the voltage of the tube.^17,18^ Iterative reconstruction technology has made significant progress, which is proved to overcome the image degradation because of tube voltage reduction.^19,20^

At the same time, the adverse reactions of iodine contrast agents and the incidence of contrast media-induced nephropathy (CIN) in intensive examinations are also important factors that plague the intensive examination.^21,22^ Reducing contrast dose is of particular interest for patients with renal dysfunction because CIN is closely related to preexisting renal insufficiency and contrast injection volume.^23^

Studies have reported the application of low-concentration iodine doses and low-tube-voltage CT in renal angiography as well as in abdominal and pulmonary nodule detection,^23–25^ but they were all single-center and single-machine studies. It was impossible to evaluate the differences in image quality caused by different CT scanners and operating technicians, resulting in the limited application of the results.

Thus, the purpose of our study is to prospectively determine, with a multicenter study, the feasibility of applying low-concentration contrast agents and low-tube-voltage CT to the examination of chest and to make it possible for its generalization and application.

## Patients and methods

### Patients

**Figure 1.**
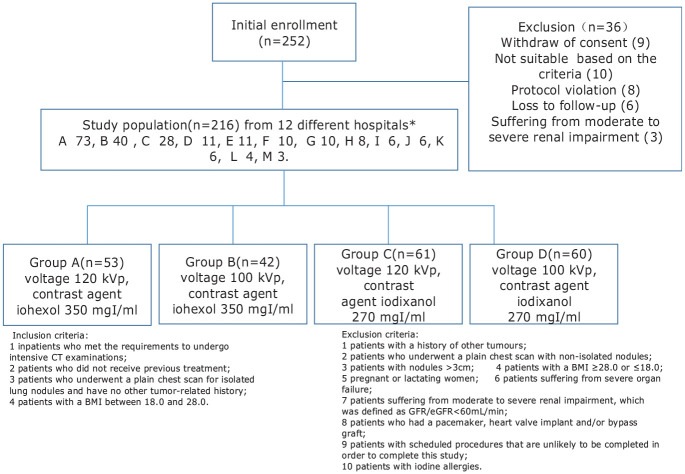
Inclusion criteria, exclusion criteria, and grouping for the enrolled study population. *Hospital centers: A: Tianjin Medical University Cancer Institute and Hospital, B: Hunan Cancer Hospital, C: Hubei Provincial Cancer Hospital, D: WISCO General Hospital, E: Hebei Cancer Hospital, F: Shanxi Cancer Hospital, G: Wuhan Third Hospital Affiliated, H: Hospital of Yan’an University, I: Hubei Huangshi General Hospital, J: Xingtai People’s Hospital, K: The Fourth Hospital of Hebei Medical University, and L: Affiliated Hospital of Inner Mongolia Medical University.

This prospective study was approved by the Medical Ethics Committee of the Tianjin Medical University Cancer Institute and Hospital. Patients with pulmonary nodules detected by plain CT and needing further diagnosis by enhanced CT were included in this study. In total, 216 inpatients with chest-enhanced CT examinations were enrolled from 12 different hospitals from 1 December 2015 to 1 July 2016. All the enrolled patients agreed to participate in the project and signed the informed consent form. The inclusion criteria, exclusion criteria, and grouping for the enrolled study population are shown in Figure 1.

### CT scanning protocols

The scanning parameters were all recommended scanning schemes. Radiologic technologists participated in this multicenter study all underwent unified and standardized training. All examinations were performed according to the recommended scanning scheme, and the scanning parameters of different machine models are listed in Table 1.

**Table 1.** Scanning parameters of different CT machine models.

	GE	SIEMENS	PHILIPS	TOSHIBA
Machine model/quantity	750/12; 64 rows/6	Dual source/3; 64 rows/7; 32 rows/1;16 rows/2	256 rows/2; 64 rows/1	320 rows/3; 64 rows/1
Group	A, C/B, D	A, C/B, D	A, C/B, D	A, C/B, D
Tube voltage	120 kVp/100 kVp	120 kVp/100 kVp	120 kVp/100 kVp	120 kVp/100 kVp
Tube current	10–400 mA	10–400 mA	10–400 mA	10–400 mA
Aperture	FOV = 40 cm	FOV = 40 cm	FOV = 40 cm	FOV = 40 cm
Reconstruction layer	1.25 mm	1.5 mm	1–2 mm	1–2 mm
Rotation time	0.6 s	0.6 s	0.6 s	0.6 s
Pitch	0.984	0.95	0.95	0.95
Noise index	14	CARE Dose 4D	Dose right	
Iterative algorithms	ASIR = 40%	SAFIR = 3	iDose = 3	AIDR3D

FOV: Field of view; ASIR: Adaptive Statistical Iterative Reconstruction; and SAFIR: Sinogramaff Irmed Iterative Reconstruction.

With the patient in the supine position, a contrast enhanced scan was performed after the plain scan. Iohexol (350 mgI/mL) or iodixanol (270 mgI/mL) was used as the contrast agent in group A and group B; iodixanol (270 mgI/mL) was used as the contrast agent in group C and group D. The injection was administered via an intravenous injection at the elbow using a high-pressure syringe at an injection rate of 2.5 mL/s. The dose was calculated at 1.5 mL/kg body weight. The scan ranging from the tip of the lung to the armpit was performed 70 s after the contrast injection. The purpose of this study was to evaluate surgical resectability, clinical stage, and efficacy evaluation. The included cases did not have the purpose of identifying benign and malignant. At 70 s, the signal density in the arterial vein reached a balanced high density, reaching a maximum density difference from the lesion. Therefore, we chose to strengthen the 70 s scan. Besides, selecting a scan of the first-stage image could reduce the dose of radiation received by the patient.

### Image analysis

Radiation and imaging data stored in digital imaging and communications in medicine (DICOM) format are exported onto a local server directly from the CT machines or via the picture archiving and communication systems used to review these examinations. Data are stripped of patient identifying information other than study date and time, and transferred to the registry.

### Subjective analysis of images

The chest CT enhancement images of the four groups of patients were independently assessed by two blinded radiologists with >10 years of experience. The mediastinal window and lung window were also analyzed. The following fixed window widths and window positions were applied: mediastinal window width, 320 HU; window level, 35 HU; and lung window width, 1500 HU; window level, −500 HU. On the mediastinal window, the readers evaluated the following four structures: trachea and paratracheal tissues (aortic arch level); carina and lymph node areas; intrathoracic esophagus (at least three levels); and pericardium (right ventricular level). The following four structures were evaluated by the viewers on the lung window: pulmonary texture, proximal bronchi and adjacent blood vessels (below the subsection level), peripheral bronchioles and adjacent blood vessels (above the subsection level), and peripheral blood vessels within 10 mm from the pleura. The following 4-point system was used for the evaluation of the images: 1 point: no noise in the image, clear structure of mediastinal window and lung window; 2 points: slight image noise, acceptable slight structural blur of mediastinal window and/or lung window; 3 points: medium image noise, acceptable medium structure blur of mediastinal window and/or lung window; and 4 points: obvious image noise, a large number of blurred shadows of mediastinal window and/or lung window, difficult to diagnose. The average mediastinal window and lung window scores were calculated separately, representing the image quality of the mediastinal window and lung window; the total average score represents the image score from 1 to 4 for the chest examination (1–3 points, can satisfy the diagnosis criteria; 4 points, cannot be diagnosed).

### Objective analysis of the images

The vascular lumen was selected at the level above the aortic window in the mediastinum window, and a region of interest (ROI) of approximately 100 mm^2^ was selected. The CT values and SD noise of the main pulmonary artery, left pulmonary artery, right pulmonary artery, thoracic paraspinal muscle, and pectoralis major muscle were measured, and the contrast-to-noise ratio (CNR) was calculated. The CNR was calculated with the following equation: CNR = (SI vascular − SI muscle)/noise, where SI blood vessels are the average CT values (HU) of the three different layers of blood vessels measured (main pulmonary artery, right pulmonary artery, and left pulmonary artery), and noise is defined as the mean of the measured ROI standard deviations (SD). The CT muscle was the average of the central position of the pectoral muscle and the attenuation of the deep paravertebral muscles on both sides.

### Evaluation of the scanning radiation dose

The volumetric CT dose index (CTDIvol) and dose length product (DLP) were recorded for each CT scan, and effective dose (ED) was calculated for each examination as follows: ED = 0.014 × DLP.

### Comparisons of imaging diagnosis accuracy

Pathological diagnosis of the patients was all recorded, and the accuracy of imaging diagnosis was compared according to the pathological results between the four groups. Imaging and pathological diagnosis must be the same in disease types such as inflammation, tuberculosis and tumor type, and site; otherwise, it was considered as nonconformity.

### Statistical analysis

The key characteristics and all measurement data were expressed as $\bar{x}\pm s$ . The objective evaluation indicators (patient data, pulmonary CT value, noise, CNR, radiation dose CTDIvol, DLP, ED) were tested by chi-square tests and one-way analysis of variance (ANOVA) using GraphPad Prism 7.0, where p < 0.05 was defined as statistically significant. The agreement of subjective evaluation between the two readers was evaluated using the weighted kappa test. The weighted kappa coefficient and the associated 95% confidence interval (CI) were calculated by MedCalc (version 15.2.2), with kappa >0.80 indicating excellent, 0.60 < kappa < 0.80 indicating good, 0.40 < kappa < 0.60 indicating moderate, and kappa < 0.40 indicating poor.

## Results

### Patient characteristics

The gender, age, height, weight, and body mass index (BMI) of the four groups of patients were not significantly different (p > 0.05), as shown in Table 2. The p values were all >0.05.

**Table 2.** Characteristics of the four groups of patients.

Group	Male/female	Age	Height (cm)	Weight (kg)	BMI (kg/m^2^)
A	35/18	60.08 ± 12.13	168.59 ± 6.96	66.51 ± 6.61	23.45 ± 2.37
B	26/16	56.38 ± 11.96	166.00 ± 7.90	64.06 ± 10.42	23.13 ± 2.51
C	42/19	61.62 ± 8.771	166.61 ± 8.10	65.89 ± 7.37	23.57 ± 3.46
D	34/26	60.7±10.2	164.75 ± 7.31	63.82 ± 7.44	23.53 ± 2.49
p value	0.5441	0.2905	0.4486	0.4095	0.8971

BMI: body mass index.

### Image quality

#### Subjective evaluation results

**Figure 2.**
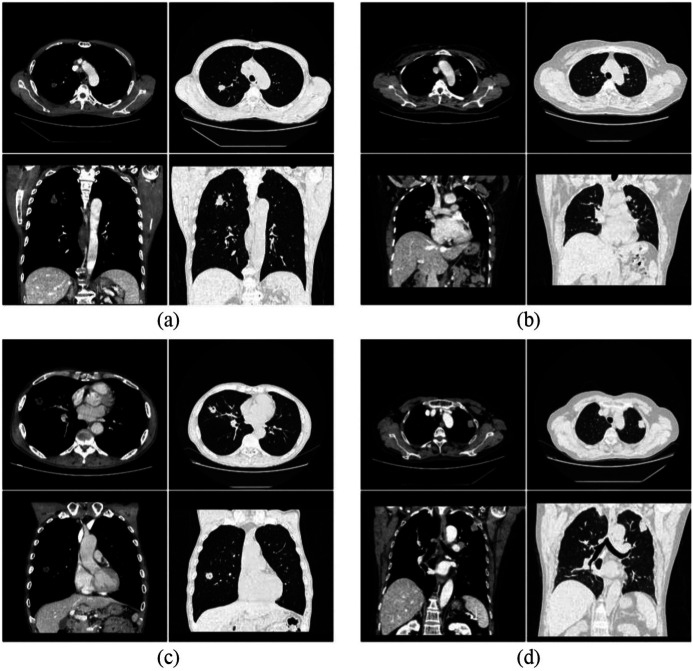
Typical pictures acquired from the four different CT scanning protocols. The patient information for the four groups was as follows: (a) male, 61, right lung upper lobe posterior segment nodules, potential diagnosis of peripheral lung cancer, peripheral inflammation. The pathological diagnosis was right upper lung invasive adenocarcinoma, and the size of the nodule was 1.8 cm × 1.6 cm; (b) female, 45, anterior medial subdural nodules of the left upper lobe, potential diagnosis of peripheral lung cancer, adhesion to the adjacent mediastinum pleura. The pathological diagnosis was cartilage-type hamartoma, and the size of the nodule was 1.3 cm × 1.9 cm; (c) male, 69, irregular nodules in the lateral segment of the right middle lobe with the surrounding military, potential diagnosis of granulomatous lesions. The pathological diagnosis was right middle lobe lung squamous cell carcinoma, and the size of the nodule was 2.0 cm × 1.5 cm; and (d) female, 63, subpleural space of the left upper lobe, potential diagnosis of peripheral lung cancer with multiple lymph node metastases. The pathological diagnosis was left lung adenocarcinoma with multiple lymph node and bone metastases.

Two experienced diagnosticians reviewed the images of all four groups of patients for image quality, and the images are shown in Figure 2 and their evaluation in Table 3. In general, the results of group A and group C were slightly better than those of group B and group D. The quality of all four groups of images could meet the diagnostic requirements, and there were no images that could not be diagnosed. The consistency of the scores by the two physicians for each of the four groups of images was good, with the kappa value ranging from 0.736 (95% CI: 0.54–0.933) to 0.809 (95% CI: 0.65–0.968).

**Table 3.** Subjective image evaluation.

Group	The score of Doctor A				The score of Doctor B				kappa	SE	95% CI
	1	2	3	4	1	2	3	4			
A	24	29	0	0	23	30	0	0	0.809	0.0812	0.650–0.968
B	10	27	5	0	9	29	4	0	0.736	0.100	0.540–0.933
C	26	35	1	0	24	36	2	0	0.778	0.0695	0.641–0.914
D	14	37	9	0	13	35	12	0	0.759	0.0729	0.616–0.902

SE: standard error; CI: confidence interval.

### Objective evaluation results

**Figure 3.**
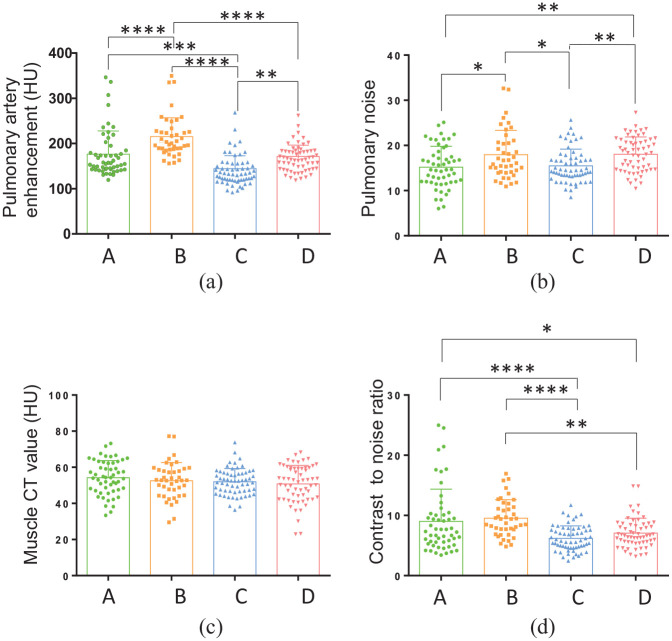
Image quality and noise comparisons of the four groups. (a) Pulmonary artery enhancement (HU), (b) pulmonary noise, (c) muscle CT value (HU), and (d) contrast-to-noise ratio were compared between the four groups. The scanning protocols were as follows: Group A: voltage, 120 kVp; iohexol contrast agent, 350 mgI/mL; Group B: voltage, 100 kVp; iohexol contrast agent, 350 mgI/mL; Group C: voltage, 120 kVp; iodixanol contrast agent, 270 mgI/mL; and Group D: voltage, 100 kVp; iodixanol contrast agent, 270 mgI/mL. *p < 0.05; **p < 0.01; ***p < 0.001; ****p < 0.0001 (one-way ANOVA).

The comparisons of image quality and noise in the four groups are shown in Table 4 and Figure 3. Groups A and B used 350 mgI/mL iodine contrast agent, so their vascular CT values were higher than those of groups C and D; Group B had the highest CT value after pulmonary artery enhancement (215.5 ± 46.1). When the voltage was 100 kVp, the noise measurements of groups B and D were higher than those of groups A and C at 120 kVp. The noise was the lowest in group A (15.2 ± 4.6), and the CNR was the best in group B (9.5 ± 3.1), followed by group A (9.0 ± 5.3), group D (7.1 ± 2.5), and group C (6.2 ± 2.1).

**Table 4.** Image quality and noise comparison.

Group	Number of cases	Pulmonary artery enhancement (HU)	Pulmonary noise	Muscle CT value (HU)	CNR
A	53	176.5 ± 51.3	15.2 ± 4.6	54.2 ± 9.4	9.0 ± 5.3
B	42	215.5 ± 46.1	17.9 ± 5.4	52.6 ± 10.0	9.5 ± 3.1
C	61	144.4 ± 33.0	15.5 ± 3.7	52.0 ± 7.3	6.2 ± 2.1
D	60	171.6 ± 29.4	18.1 ± 3.8	50.8 ± 10.3	7.1 ± 2.5
F value		26.24	6.78	1.343	11.27
p value		<0.0001	0.0002	0.2615	<0.0001

CT: computed tomography; CNR: contrast-to-noise ratio.

### Comparison of effective radiation measurements

**Figure 4.**
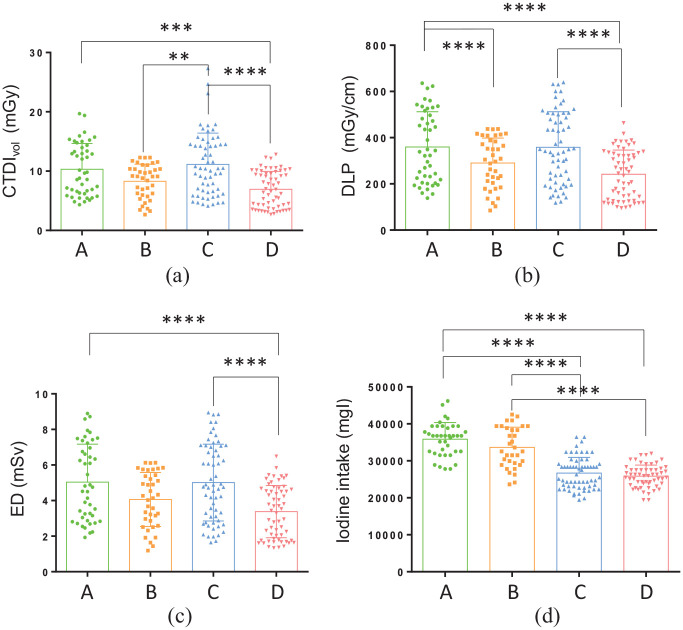
Comparisons of scanning radiation doses of the four groups. (a) CTDI_vol_ (mGy), (b) DLP (mGy/cm), (c) ED (mSv), and (d) iodine intake (mgI) were compared between the four groups. The scanning protocols were as follows: Group A: voltage, 120 kVp; iohexol contrast agent, 350 mgI/mL; Group B: voltage, 100 kVp; iohexol contrast agent, 350 mgI/mL; Group C: voltage, 120 kVp; iodixanol contrast agent, 270 mgI/mL; and Group D: voltage, 100 kVp; iodixanol contrast agent, 270 mgI/mL. CTDIvol: volumetric CT dose index, DLP: dose length product, ED: effective dose. *p < 0.05; **p < 0.01; ***p < 0.001; ****p < 0.0001 (one-way ANOVA).

The comparisons of the scanning radiation dose are shown in Table 5 and Figure 4. The difference between the groups was relatively large and statistically significant (p < 0.05). In the four groups, patients in groups B and D had lower EDs than patients in groups A and C, with reductions of 19% (0.975 mSv) and 33% (1.654 mSv), respectively. The patients in group D had the lowest iodine intake.

**Table 5.** Comparison of scanning radiation dose.

Group	Number of cases	CTDI_vol_ (mGy)	DLP (mGy/cm)	ED (mSv)	Iodine intake
A	53	10.3 ± 4.4	359.7 ± 152.7	5.0 ± 2.1	35,835 ±4500
B	42	8.3 ± 2.9	290.1 ± 108.3	4.1 ± 1.5	33,632 ± 5472
C	61	11.2 ± 5.3	358.2 ± 154.0	5.0 ± 2.2	26,670 ±4283
D	60	6.9 ± 3.0	241.6 ± 104.7	3.4 ± 1.5	25,848 ±3014
F value		12.44	10.36	10.36	62.33
p value		<0.0001	<0.0001	<0.0001	<0.0001

CTDI_vol_: volume CT dose index; DLP: dose length product; ED: effective dose.

### Comparison of coincidence rate between CT diagnosis and pathological diagnosis

All four groups were followed up with pathological diagnoses. Of the 216 cases, 181 cases were diagnosed by surgical resection or biopsy, including 91 cases of adenocarcinoma, 43 cases of squamous cell carcinoma, 12 cases of small cell lung cancer, and 12 cases of granulomatous lesions. Besides, five cases of hamartoma, nine cases of inflammatory pseudotumors, six cases of chronic inflammatory cell infiltration, two cases of schwannomas, and one case of sclerosing hemangioma were included. There were no pathological results in 35 of the cases. There were no significant differences in the imaging diagnosis accuracy rate between the four groups (p > 0.05), and the data are shown in Table 6.

**Table 6.** Comparison of concordance rate between CT diagnosis and pathological diagnosis.

Group	Number of cases	Pathological diagnosis	Imaging diagnosis	Accuracy rate (%)
A	53	45	38	84.4
B	42	36	30	83.3
C	61	50	42	84
D	60	50	41	82
F value	0.085			
p value	0.994			

## Discussion

This is a multicenter study using different CT models with different operators but the same protocol to examine different areas of the patient population, so the relevant same imaging physician-read parameters can better evaluate the applicability of low-concentration contrast agents and low-tube-voltage CT examinations.

Studies have shown that lowering the tube voltage can significantly reduce the patient’s radiation dose,^19,26,27^ but the tube voltage is not as low as possible, the better, it is necessary to choose the appropriate voltage according to the patient’s characteristics (gender, age, height, weight, and BMI). Research found that there was no significant difference between the subjective evaluation and objective parameters of image quality in the routine chest CT examination using 100 or 120 kV.^18,28^ Therefore, a tube voltage of 100 kVp was selected in this study and there was no significant difference in patient’s characteristics. In addition, it was reported that the use of the isotonic contrast agent iodixanol leads to a reduced risk of CIN compared to low osmolal contrast agents.^29,30^ It can be seen that the choice of contrast agent concentration is closely related to the subsequent contrast agent reaction. From the perspective of reducing the amount of iodine contrast agent to reduce the incidence of adverse reactions, we used iohexol (350 mgI/mL) and iodixanol (270 mgI/mL) for comparison.

Lowering the tube voltage could reduce the penetration of X-rays and increase their attenuation after reaching the organ. It is suitable to lower the tube voltage due to the high intrinsic contrast between the gas-filled lung and soft tissue.^31,32^ Moreover, reducing the tube voltage can make the output X-ray energy closer to the K-edge of iodine (33 keV). Therefore, the detection efficiency of the iodine signal can be improved, so the CT value of the iodine contrast agent can be increased.^28,33^ Our study showed that using same concentration contrast agent, the CT value of group B (215.5 ± 46.08 HU) at a tube voltage of 100 kVp was 18.1% higher than that of group A (176.5 ± 51.33 HU) at 120 kVp; the value of group D (171.6 ± 29.4 HU) at a tube voltage of 100 kVp was 15.9% higher than that of group C (144.4 ± 33.04 HU) at 120 kVp. However, compared with group A, group D showed no difference in CT values, mainly because the increase in CT value that was caused by lowering the tube voltage can be compensated by lowering the dosage of iodine agent.^34^

Meanwhile, low-tube-voltage scan protocol was associated with an increase in image noise,^35^ because the penetrating power is reduced, but the image contrast can be improved. The image noise was higher in group B (17.9 ± 5.4) than in group A (15.2 ± 4.6), with an increase of 15.1%, and compared with group C (15.5 ± 3.7), the noise of group D (18.1 ± 3.8) was increased by 14.4%, consistent with Nakaura et al.,^19,36^ who reported that noise of the low-voltage group was much higher than that of the high-voltage group. However, when the tube voltage is the same, the change in contrast agent concentration has no significant effect on noise. Studies have shown that reducing tube voltage in combination with iterative reconstruction techniques has no significant effect on CNR compared to conventional inspection during CT examination.^37,38^ Combined with the results of our study, CNR is related to the concentration of contrast agent and has no obvious relationship with tube voltage. Ultimately, there was no difference in the subjective evaluations of the two groups of images.

CTDI_vol_ reflects the average dose value within a section (slice) of the scanned volume, whereas DLP reflects the total emitted radiation imparted to the patient. ED, which is proportional to the total imparted radiation, is an estimate that accounts for estimated future cancer risk based on irradiated organs. Besides, the radiation dose is proportional to the square of the tube voltage.^15^ Feuchtner et al. compared 100 kVp with 120 kVp for routine chest CT examination. The radiation dose of 100 kVp is significantly lower than that of 120 kVp, which reduces by 15%–48%.^39^ Our study showed that the radiation dose of group B (4.1 ± 1.5 mSv) using 100 kVp was reduced by 18% compared with that of group A with 120 kVp (5.0 ± 2.1 mSv), and the ED of group D (3.4 ± 1.5 mSv) using 100 kVp was lower by 32% compared with that of group C (5.0 ± 2.2 mSv) with 120 kVp. Recent research indicated that the largest driver of dose variation was how providers or clinical staff chose to set the machine technical parameters, not the machine.^40^ Our research results also prove this.

There are also some limitations to the current study: although this is a multicenter study, the number of cases included is relatively small. To eliminate the confounding factors, we limited the research to patients with BMI between 18 and 28. More data need to be collected to promote the application of these results.

In enhanced CT examinations, there is a balance among image quality, the amount of iodine injected, and radiation dose.^23^ Notably, the images of group D that were acquired with the protocol with a voltage of 100 kVp and iodixanol contrast agent (270 mgI/mL) had no differences between the objective and subjective evaluations compared with the images of conventional group A (voltage, 120 kVp; iohexol contrast agent, 350 mgI/mL); not only could these images meet the diagnostic requirements but also the radiation dose was reduced by 32%. Therefore, the main factor affecting CT image quality and the evaluation results was the scanning protocol, not the operator, machine manufacturer, or machine model.

## Conclusion

The protocol with low-concentration contrast agents (270 mgI/mL) and low-tube-voltage (100 kVp) CT can not only decrease a certain degree of radiation dose but also guarantee the image quality and meet the needs of imaging diagnosis in chest enhancement examinations, which makes it possible for its generalization and application.
